# Compatibility Assessment of Novel Orodispersible Film Vehicle for Personalized Medicine with Selected Active Pharmaceutical Ingredients

**DOI:** 10.3390/jpm13111565

**Published:** 2023-10-31

**Authors:** Hudson C. Polonini, Anderson O. Ferreira, Nádia R. B. Raposo, Paulo José L. C. da Silva, Marcos Antônio F. Brandão

**Affiliations:** 1 Fagron BV, Fascinatio Boulevard 350, 3065 WB Rotterdam, The Netherlands; anderson.ferreira@fagron.com; 2 Research and Innovation Center for Health Sciences, Federal University of Juiz de Fora (UFJF), Juiz de Fora 36038, MG, Brazil; nadia.barbosa@ufjf.br (N.R.B.R.); pjcandido@gmail.com (P.J.L.C.d.S.); marcos.brandao@ufjf.br (M.A.F.B.)

**Keywords:** orodispersible films, personalizing medicine, compatibility, stability, compounding pharmacy

## Abstract

Orodispersible films (ODFs) are solid pharmaceutical forms for rapid local or systemic release of active ingredients. They are formed by a water-soluble polymer film that hydrates rapidly, adhering and dissolving immediately when placed on the tongue or in the oral cavity. In this paper, we describe the compatibility and disintegration times of compounded ODFs using OrPhyllo^TM^, a new ready-to-use-vehicle, and APIs from different pharmacological classes, such as 5-hydroxytryptophan (5-HTP) 50 mg, bromopride 5 mg, coenzyme Q10 20 mg, melatonin 3 mg, resveratrol 5 mg, tadalafil 10 mg, vitamin B12 1 mg, or vitamin D3 2000 UI. ODFs were compounded and, subsequently, the samples were assayed using HPLC at initial (t = 0), 7 days (t = 7), 14 days (t = 14), 30 days (t = 30), 60 days (t = 60), 90 days (t = 90), 120 days (t = 120), 150 days (t = 150), and 180 days (t = 180) after compounding. Given the percentage of recovery of the APIs within the films, the beyond-use date of the final products (API + vehicle) was at least 90 days for vitamin D3, 150 days for bromopride and 5-HTP, and 180 days for coenzyme Q10, tadalafil, vitamin B12, resveratrol, and melatonin, when stored at room temperature. The average disintegration time was 46.22 s. This suggests that the OrPhyllo^TM^ vehicle is suitable for compounding ODFs with APIs from different pharmacological classes, with good compatibility and fast disintegration.

## 1. Introduction

Orodispersible films (ODFs) are innovative solid pharmaceutical dosage forms intended to convey and quickly release active pharmaceutical ingredients (APIs) or food supplements locally or systemically. They are formed by a water-soluble polymeric film that moisturizes quickly, adhering and dissolving immediately when placed on the tongue or the oral cavity (e.g., buccal, palatal, gingival, lingual, or sublingual), with no need for water or chewing [1,2,3,4,5]. Although ODF is the official term used by the European and the British Pharmacopoeias [6,7], they can also be found on the market as oral, mouth, fast dissolving, mouth dissolving, oral soluble, or oral transmucosal film, as well as oral strip. The United States Pharmacopeia refers to the dosage form as “films”, which are subdivided into oral films (delivering medication to the mouth or to the gastrointestinal tract for absorption), and buccal films and sublingual films (which “facilitate absorption through the proximal mucosal membranes avoiding first pass metabolism or degradation in the gastrointestinal tract and providing a quick onset of action”) [8].

A vast body of evidence shows that ODFs are suitable for a broad range of therapeutic purposes. For instance, analgesics, anti-asthmatics, anticonvulsants, antiemetics, antihistamines, antihypertensives, anxiolytics, diuretics, anti-inflammatories, antiseptics, sedatives, etc. [2,9,10,11]. Additionally, they are ideal for personalized treatments for specific groups, such as pediatrics, geriatrics, and patients with dysphagia, allergies, or dietary restrictions [12]. Therefore, ODFs can be understood as patient-centered pharmaceuticals beneficial to all patient groups [10,11,13].

There are many processes for producing ODFs: hot-melt extrusion, solvent-casting, freeze-drying, rolling methods, and 3D extrusion printing [14,15,16,17]. Most of them demand high investments in equipment and are adequate to produce large batches of a single product in a single strength. However, the abovementioned methods are not adequate for compounding pharmacy settings. Given this, we present a complete solution to produce ODFs in compounding pharmacies to overcome the limitations, using the laminating process (solvent-casting method), as it is the one that provides the best accuracy and content uniformity for compounding pharmacies’ scale. To the best of the authors’ knowledge, our group was the first to discuss this technology for compounding pharmacies, in 2017 [4,5].

This solution to produce films comprises a new ready-to-use vehicle for ODFs (OrPhyllo^TM^, Fagron, The Netherlands) and the necessary equipment to produce them safely, accurately, and in a suitable scale for personalization (a laminating device and a film dryer, both from Fagron). To show the functionality of this set, we have compounded eight different ODF formulations and evaluated their organoleptic characteristics, average weight, disintegration, content uniformity, and stability. The APIs evaluated were coenzyme Q10 (ubidecarenone, a coenzyme in mitochondrial electron transport; used for support energy production), tadalafil (selective inhibitor of cyclic GMP-specific phosphodiesterase with vasodilator action; treatment of erectile dysfunction), vitamins B12 and D3, resveratrol (polyphenolic phytoalexin; antioxidant), melatonin (regulates the normal sleep/wake cycle), bromopride (dopamine antagonist; antiemetic) and 5-hydroxytryptophan (an amino acid used for sleep disorders) [18]. The aim was to assess whether the ODFs are a feasible option for those APIs in the daily routine of a compounding pharmacy.

## 2. Materials and Methods

### 2.1. Formulation and Preparation of the Films

The products evaluated throughout the study are described in Table 1.

All APIs were obtained from Fagron and met United States Pharmacopeia (USP) and European Pharmacopeia grades. Previously to the compounding of the finished films, the OrPhyllo^TM^ film base was reconstituted in the following manner: 22.5 g of the OrPhyllo^TM^ film base was transferred to a mortar and dispersed with 50 g of purified water (solvent); after that, 1 g of polysorbate 80 (surfactant), 3.75 g of polyethylene glycol 400 (plasticizer), 1.0 g of simethicone 7-9245 emulsion 30% (adjuvant to decrease bubble formation), and water q.s. 100 g were added and mixed until homogeneity. This was stored in amber glass bottles for up to 3 months before use.

**Figure 1:**
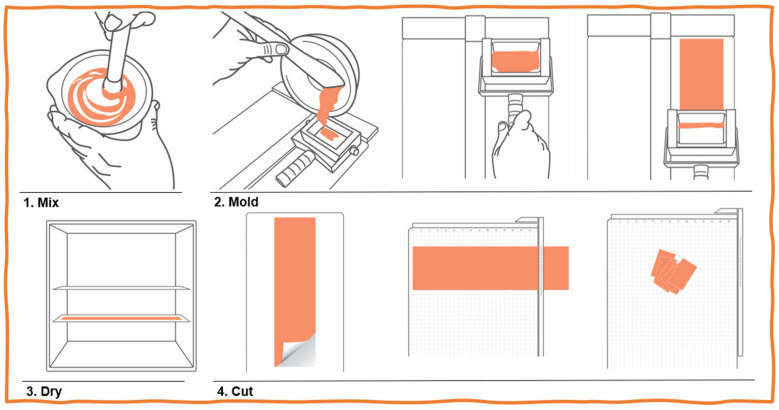
Compounding steps for orodispersible films: (1) mix APIs with OrPhyllo^TM^ and liquid components; (2) transfer the contents to the laminating apparatus and spread the contents over a glass plate (different designs with and without the handle are available); (3) dry the films under hot air in a film dryer; and (4) cut the films in the desired size. This laminating process is the most adequate for compounding pharmacies to produce films with adequate content uniformity.

The compounding process of the finished films included the following 4 steps (Figure 1):

1. All APIs were sieved, crushed in a mortar, and then carefully mixed with the Orphyllo^TM^ until a paste was obtained.
2. This mixture was laminated/spread onto glass plates using a laminating apparatus (FagronLab).
3. The glass plates were transferred to a film dryer (FagronLab) previously equilibrated at 40 °C for 40 min.
4. After completely drying, films were cut with a guillotine (paper trimmer) into squares of 3 × 3 cm, packed into individual laminated matte aluminum sachets, and stored at noncontrolled room temperature to mimic real-use conditions (15–30 °C).

### 2.2. Compatibility Study

ODFs were assayed by high-performance liquid chromatography (HPLC) at pre-determined time points to verify the compatibility of the API in the vehicle. This protocol followed general guidelines from USP for estimating beyond-use dates (BUDs) and previous works conducted by our research group [19,20,21]. Briefly, samples were diluted in the mobile phase to obtain work solutions in the concentration described in Table 2. Sampling times were: initial (t = 0), 7 days (t = 7), 14 days (t = 14), 30 days (t = 30), 60 days (t = 60), 90 days (t = 90), 120 days (t = 120), 150 days (t = 150), and 180 days (t = 180). All samples were assayed six times, and the results were expressed as the mean from six independent measurements. Before injection into the HPLC, samples were filtered in 15 mm regenerated cellulose syringe filters with 0.45 μm pore size. The evaluation parameter was the percent recovery to t = 0, using the HPLC method (results given as percentage ± standard deviation).

Analyses were performed following the methods described in Table 2 on a qualified and calibrated chromatography system (Young Lin, Anyang, Republic of Korea). This is composed of a quaternary gradient pump (YL 9110), a photodiode array (PDA) detector (YL 9160), a 96-vial programmable autosampler (YL 9150), a column oven compartment (YL 9130), a variable sample loop up to 200 μL and a software controller (Clarity version 8.1). The columns were connected with a pre-column with the same packing (4.0 × 3.0 mm, 5 μm) from the same vendor of the columns.

Methods were evaluated concerning their stability-indicating characteristics. API samples were subjected to the following stressing conditions to determine the capacity of the HPLC method to detect any possible degradation products that may arise during storage of the oral films: (i) dilution in acid (0.1 M HCl, at 25 °C); (ii) dilution in base (0.1 M NaOH, at 25 °C); (iii) exposure to ultraviolet light at 365 nm (at 25 °C); (iv) heating at 70 °C; and (v) dilution in H_2_O_2_ 35% (*v*/*v*) (at 25 °C).

### 2.3. Disintegration

Melatonin 3 mg, bromopride 5 mg, and 5-HTP 50 mg films were evaluated for their disintegration time using a disintegrator (301AC, Ethik, São Paulo, Brazil) and following the general guidelines from the USP and the British Pharmacopeia, with adaptations due to the lack of specific requirements for ODFs [22,23]. Briefly, a water tub was stabilized within the temperature range of 37 ± 1 °C maintained by a thermostat. Next, a 1 L beaker containing purified water obtained in reverse osmosis was immersed in this water tub. The assay consists of a compartmentalized basket divided into six tubes, where this basket is immersed and removed from the beaker at a constant velocity. This immersion occurs through an automated mechanism programmed to move vertically at a constant speed, upwards and downwards. For experimental purposes, one film was arranged in each of the six basket tubes and a plastic disc was added to each tube to fix the film on the tube base. The movement of the mechanism stopped after 1 min and the disintegration time was taken before the device was turned off.

## 3. Results and Discussion

ODFs are pharmaceutical dosage forms that account for numerous advantages, such as (i) fast disintegration and dissolution in the oral cavity for local action or systemic absorption of the API; (ii) increased bioavailability, as some APIs can be absorbed from the mouth (including sublingual), pharynx, and esophagus as the saliva is swallowed and reaches the stomach; (iii) quick start of action (meaningful in motion sickness, acute pain, cough, or allergic episodes, where the rapid start of action is required); (iv) no risk of choking or suffocation; (v) ease of administration in dysphagic, pediatric, geriatric, and psychiatric patients refusing to swallow tablets or capsules; (vi) do not require water or mastication for their administration (a convenient situation for patients who are traveling or with nausea and vomiting); (vii) flexible and portable (minimum volume, light, and small), facilitating transportation, use, and storage [3,14].

Despite the previously mentioned advantages of the ODFs, some caution regarding dosage uniformity and stability of the compounded products should be addressed. For dosage uniformity, Raposo et al. [24] have already shown that the compounding method presented here is accurate, as it is possible to obtain content uniformity for the films. The work also divided each film into four pieces and showed that the drug distribution is homogeneous in all parts of the films. It is important to highlight this, as other methods previously accessed by our team during the development phase showed that processes such as using molds to cast the films might lead to the ununiform distribution of the API throughout the film surface, with a deposit on the edges—this creates a non-ideal buccal sensation for the patient, together with a risk of failing content uniformity testing.

Moreover, it is important to note that the OrPhyllo^TM^ base is a commercial product with a validated shelf-life of two years, as determined through comprehensive stability studies that encompass both physico-chemical and microbiological assessments. It is formulated using pullulan and xanthan gum, both of which have been designated as Generally Recognized as Safe (GRAS) ingredients by the United States Food and Drug Administration (FDA) [25]. OrPhyllo^TM^ is also vegan, meaning it is not expected to contain Bovine Spongiform Encephalopathy (BSE) or Transmissible Spongiform Encephalopathy (TSE) agents. Furthermore, it is free from parabens, phthalates, propylene glycol, and alcohol, underscoring the safety profile of this base.

Concerning compatibility, we present data supporting a suitable BUD for the films. Studying the physicochemical compatibility of the compounded formulations is paramount to preventing non-homogeneous dosing, which can lead to medication errors. The final and stable film is a product of multiple factors, including the API’s chemical, physical, and microbiological stabilities, and interaction with the vehicle and the packaging. In this study, we evaluated the compatibility of selected APIs in the ODFs as a factor of degradation of the API with time.

The APIs studied were coenzyme Q10, tadalafil, vitamins B12 and D3, resveratrol, melatonin, bromopride, and 5-hydroxytryptophan. The doses chosen for these APIs were based on several factors: available scientific literature, safety profiles, therapeutic ranges, and practical considerations. It is worth noting that dosage forms like ODFs have limited space for the active ingredient, which can influence the chosen dosage. For 5-HTP, often used to support serotonin production and mood regulation, the dosage of 50 mg falls within the lower end of the typical range (50–200 mg). Bromopride is commonly prescribed at doses ranging from 10 to 20 mg for gastrointestinal issues, so 5 mg is at the lower end of this range because at this concentration it can be used for pediatric patients. The same applies to coenzyme Q10 at 20 mg. The dosage of 3 mg/ODF for melatonin falls within its common range. For resveratrol at 5 mg/ODF, the dosage is relatively low compared to typical supplements, but this is justified because it is being studied for its use as a long-term supplement and the orodispersible route may have a positive impact on drug absorption. Tadalafil is typically used to treat erectile dysfunction and 10 mg is a common starting dose. The dosage of 1 mg for vitamin B12 is generally considered sufficient for individuals with a deficiency in vitamin B12. The dosage of vitamin D3 is within the typical range for daily supplementation, which can vary from 600 to 2000 IU or more depending on an individual’s needs and existing levels.

It is noteworthy that all tested APIs were sensitive for all the stressing conditions tested, except resveratrol when exposed to UV light (as it is a polyphenol that can absorb these wavelengths and act as a sunscreen agent), and vitamin B12 when exposed to UV light and heating (Table 3). This shows that those are sensitive APIs; therefore, a good selection for evaluating the new method and vehicle is described here. All other conditions lead to significant decomposition. Once the forced-degradation profiles of the APIs were determined, which confirmed the suitability of the method for the compatibility study, the chemical compatibility of the ODFs was evaluated.

The chemical compatibility results are shown in Table 4 and are expressed as a relative percentage of recovery (initial sampling time = 100%). For the products to be stable, the relative percentage recovery should lie within 90–110%. Although sampling times were different for the APIs, all products remained stable throughout the study (at least 90 days for vitamin D3, 150 days for 5-HTP, and 180 days for bromopride, coenzyme Q10, melatonin, resveratrol, tadalafil, and vitamin B12, when stored at room temperature).

To the best of the authors’ knowledge, there is no report in the literature dealing with the compatibility of such APIs in ODFs. The lack of this data highlights the importance of this study, and its innovative approach demonstrates the formulation’s effectiveness. For example, although the USP provides a BUD limit of 180 days for nonaqueous dosage forms with water activity lower than 0.60, they also state that a shorter BUD must be assigned when the physical and chemical stability of the compounded nonsterile preparation is less than the BUD suggested [19]—the lower BUD observed here for vitamin D3 ODFs is a good example of such approach.

The disintegration assay demonstrated that the formulation (vehicle) for the APIs was suitable for the desired application. The observed disintegration times were 47.8 s for melatonin 3 mg, 46.7 s for bromopride, and 46.2 s for 5-HTP 50 mg (global average = 46.94 s + 0.83 s). Thus, it confirms the fast-dissolving property of ODFs. The disintegration time of ODFs typically exhibits brevity, frequently falling within a range of seconds to a few minutes. As an illustrative instance, El-Setouhy and El-Malak observed in vitro disintegration times of less than 60 s for newly developed tianeptine sodium ODFs [26]. The primary objective of this rapid disintegration characteristic is to ensure prompt dissolution or disintegration within the oral cavity, offering enhanced convenience, especially for patients encountering difficulties in swallowing conventional tablets or capsules. This rapid disintegration feature is a pivotal attribute of ODFs, with the potential to bolster patient adherence and augment the overall therapeutic efficacy of the administered medication. While there is no universally stipulated temporal threshold defining the disintegration time for all ODFs, it is commonly recognized that this duration should typically fall within a maximum limit of 3 min [27]. Manufacturers routinely undertake systematic investigations to determine the precise disintegration time for their specific ODF formulations, with the dual objective of meeting regulatory expectations and optimizing the patient experience and therapeutic outcomes. The specific disintegration time can exhibit considerable variation among distinct ODF products, contingent upon a plethora of formulation and manufacturing factors [28,29].

## 4. Conclusions

The proposed method (lamination; solvent-casting) to produce personalized ODFs in a compounding pharmacy setting showed promising results. Disintegration rates were fast enough to suggest a suitable quick start of effects in vivo. Additionally, compatibility tests showed that the BUDs ranged from 90 to 180 days when stored at room temperature (90 days for vitamin D3, 150 for 5-hydroxytryptophan, and 180 for bromopride, coenzyme Q10, melatonin, resveratrol, tadalafil, and vitamin C). This suggests that ODFs using OrPhyllo^TM^ as a vehicle and the laminating production process represent a pharmaceutical form suitable for compounding APIs from different pharmacological classes, and have good compatibility and fast disintegration into the oral cavity.

## Figures and Tables

**Table 1:** Composition of the orodispersible films evaluated.

API	Quantity per Film	
	API	OrPhyllo^TM^ (Base, Reconstituted)
5-Hydroxytryptophan (5-HTP)	50 mg	qs 1 film
Bromopride	5 mg	qs 1 film
Coenzyme Q10	20 mg	qs 1 film
Melatonin	3 mg	qs 1 film
Resveratrol	5 mg	qs 1 film
Tadalafil	10 mg	qs 1 film
Vitamin B12	1 mg	qs 1 film
Vitamin D3	2000 UI	qs 1 film

qs = quantity sufficient for.

**Table 2:** Chromatographic conditions were used for quality control of the films.

API	Mobile Phase Composition	Work Concentration (μg/mL) *	Column	Flow (mL/min)	UV Detection Wavelength (nm)
5-HTP	Methanol 3% in 0.05 M phosphate buffer	50.0; 20 μL injection	C18, 4.6 mm × 25 cm, at 25 °C	1.5	275
Bromopride	Phosphate buffer pH 7.0 and acetonitrile (60:40, *v*/*v*)	80.0, in water and acetonitrile (3:2); 20 μL injection	L11, 4.6 mm × 25 cm, at 25 °C	1.0	310
Coenzyme Q10	Methanol and ethanol (65:35, *v*/*v*).	1000.0, in ethanol; 10 μL injection	C18, 4.6 mm × 15 cm, at 35 °C	1.0	275
Melatonin	Acetonitrile and phosphate buffer pH 3.5 (25:75, *v*/*v*)	30.0; 20 μL injection	C18, 4.6 mm × 15 cm, at 25 °C	1.0	222
Resveratrol	Acetonitrile and water (55:45, *v*/*v*)	50.0; 20 μL injection	C18, 4.6 mm × 25 cm, at 25 °C	1.4	307
Tadalafil	Acetonitrile, water, and trifluoroacetic acid (35:65:1, *v*/*v*/*v*)	100.0, in water; 20 μL injection	C18, 4.6 mm × 15 cm, at 35 °C	1.0	285
Vitamin B12	Methanol and water (7:13, *v*/*v*)	5.0, in water; 100 μL injection	C18, 4.6 mm × 15 cm, at 25 °C	0.5	361
Vitamin D3	Water and methanol (3:97, *v*/*v*)	5.0, in methanol 90%; 100 μL injection	C18, 4.6 mm × 10 cm; at 25 °C	1.0	264

* Diluted in the mobile phase unless stated otherwise.

**Table 3:** Summary of the stability-indicating study for the active pharmaceutical ingredients (APIs) evaluated.

API	HCl	NaOH	UV	Heat	H_2_O_2_
	%d *	%d *	%d *	%d *	%d *
5-HTP	**−14.18**	**−13.03**	**−13.63**	**−4.17**	**−2.96**
Bromopride	**−5.90**	**−15.35**	**−8.78**	**3.65**	**−5.67**
Coenzyme Q10	**−98.45**	**ND**	**ND**	**−29.69**	**38.77**
Melatonin	**−93.03**	**63.19**	**−44.51**	**−4.17**	**2.01**
Resveratrol	**−81.38**	**−91.31**	1.82	**−12.88**	**ND**
Tadalafil	**13.72**	**9.98**	**13.29**	**9.68**	**54.57**
Vitamin B12	**−12.59**	**−96.40**	−1.86	−1.70	**12.44**
Vitamin D3	**−2.48**	**−4.99**	**−51.88**	**−46.89**	**65.19**

* %d = percentage of discrepancy between the API peak without submission to stressing factors (negative control) and the peak of a sample subjected to one of the cited accelerated-degradation factors. ND = non-detected. Areas are given as mV. Maximum acceptable = 2% (values higher than this are in bold).

**Table 4:** Compatibility of the APIs in the compounded oral dispersible films.

Elapsed Time (Days)	% Recovery (Mean ± Standard Deviation; *n* = 6)
	Room Temperature (15–30 °C)
5-HTP—50 mg/oral film	
t = 0	100.00 ± 2.99
t = 30	101.34 ± 2.10
t = 60	101.16 ± 3.33
t = 90	97.06 ± 1.78
t = 120	98.70 ± 1.43
t = 150	101.95 ± 2.92
Bromopride—5 mg/oral film	
t = 0	100.00 ± 2.82
t = 30	95.42 ± 5.99
t = 60	97.36 ± 2.01
t = 90	97.53 ± 2.20
t = 120	96.65 ± 2.03
t = 150	97.03 ± 1.87
t = 180	109.72 ± 5.10
Coenzyme Q10—20 mg/oral film	
t = 0	100.00 ± 3.83
t = 7	100.79 ± 5.54
t = 14	103.95 ± 2.38
t = 30	102.31 ± 3.14
t = 60	106.15 ± 4.51
t = 90	102.61 ± 2.38
t = 120	103.17 ± 4.84
t = 150	104.37 ± 4.87
t = 180	104.81 ± 2.94
Melatonin—3 mg/oral film	
t = 0	100.00 ± 1.80
t = 30	100.50 ± 2.91
t = 60	97.96 ± 2.50
t = 90	97.21 ± 3.53
t = 120	100.56 ± 4.60
t = 150	101.30 ± 4.96
t = 180	93.56 ± 2.63
Tadalafil—10 mg/oral film	
t = 0	100.00 ± 2.52
t = 7	99.28 ± 4.38
t = 14	102.47 ± 2.73
t = 30	96.46 ± 1.36
t = 60	95.67 ± 2.76
t = 90	95.83 ± 1.08
t = 120	98.16 ± 0.93
t = 150	92.23 ± 4.83
t = 180	91.47 ± 2.63
Resveratrol—5 mg/oral film	
t = 0	100.00 ± 3.16
t = 7	100.72 ± 5.90
t = 14	101.29 ± 4.21
t = 30	100.56 ± 1.01
t = 60	99.21 ± 1.53
t = 90	96.37 ± 2.26
t = 120	96.21 ± 3.31
t = 150	98.24 ± 1.79
t = 180	99.04 ± 3.67
Vitamin B12—1 mg /oral film	
t = 0	100.00 ± 4.03
t = 7	102.40 ± 2.30
t = 14	101.78 ± 1.26
t = 30	101.32 ± 3.06
t = 60	102.17 ± 1.55
t = 90	100.71 ± 1.68
t = 120	99.69 ± 2.41
t = 150	99.85 ± 2.69
t = 180	95.47 ± 1.96
Vitamin D3—2000 UI/oral film	
t = 0	100.00 ± 4.17
t = 7	102.16 ± 4.68
t = 14	102.96 ± 4.61
t = 30	102.85 ± 4.18
t = 60	100.70 ± 4.26
t = 90	102.27 ± 3.72

## Data Availability

The data presented in this study are available on request from the corresponding author. The data are not publicly available due to non existence of repository for chromatograms for such studies.
